# Whole-Genome Analysis of Cervical Human Papillomavirus Type 35 from rural Zimbabwean Women

**DOI:** 10.1038/s41598-020-63882-z

**Published:** 2020-04-24

**Authors:** Megan B. Fitzpatrick, Zoe Hahn, Racheal S. Dube Mandishora, Jenny Dao, Jenna Weber, ChunHong Huang, Malaya K. Sahoo, David A. Katzenstein, Benjamin A. Pinsky

**Affiliations:** 1 Stanford Health Care, Stanford, CA USA; 2 Stanford University School of Medicine, Department of Pathology, Stanford, CA USA; 3 University of Zimbabwe College of Health Sciences, Department of Medical Microbiology, Harare, Zimbabwe; 4 Stanford University School of Medicine, Department of Medicine, Division of Infectious Diseases and Geographic Medicine, Stanford, CA USA

**Keywords:** Molecular medicine, Cervical cancer, Infection

## Abstract

Human papillomavirus (HPV) types differ by geographic location and the ethnicity of the human host, which may have implications for carcinogenicity. HPV35 is one of the least frequently identified high-risk types in North America and Europe but was the most common high-risk HPV (hrHPV) infection in a cohort in rural Zimbabwe. Whole genome analysis is limited for HPV35; no such studies have been performed in Zimbabwe. Of 648 women in the initial cohort in Zimbabwe, 19 (19/648, 2.9%) tested positive for HPV35, and eight samples were successfully sequenced for HPV35. The maximum number of sequence variants for the whole genome was 58 nucleotides (0.7%) compared to the prototype (58/7879). The maximum number of sequence variants in E6 and E7 was 3 (3/450, 0.7%) 2 (2/300, 0.7%), respectively. These are the first HPV35 whole genome sequences from Zimbabwe, and these data further lend support to the carcinogenicity of HPV35 despite limited sequence heterogeneity. Further studies to determine carcinogenic effects and impact of HPV vaccinations are warranted, especially in sub-Saharan Africa.

## Introduction

Human Papillomaviruses (HPV) are circular double-stranded DNA viruses known to infect humans. The genome is approximately 7,900 base pairs and is characterized by early genes (E1, 2, 4–7), late genes (L1-2), long control region (LCR), and a variable non-coding region (between E5 and L2)^1^. More than 200 HPV types have been identified, categorized by genetic structure and mucosal tropism^2^. The *Alphapapillomavirus* genus primarily infects human mucosal surfaces, and the types therein are classified into clades and low-risk, intermediate-risk (or possible, probable carcinogenic) and high-risk types, depending on their carcinogenic potential^3^.

Most cervical cancers are caused by HPV16 and HPV18, together comprising ~70% of cervical cancers^4^. Human papillomavirus 16 (HPV16) is classified in clade alpha-9, along with other carcinogenic types including HPV31, 33, 35, 52, and 58^5^. Among these types, HPV35 is one of the least frequently identified in North America and Europe but is commonly identified in studies from sub-Saharan Africa^6,7^. For example, studies in Mozambique found HPV35 to be the most common hrHPV type among hrHPV-positive women and women with cervical neoplasia^8^, as well as the fourth most frequently detected type in cervical cancers^9^. Similarly, work from Malawi found HPV35 to be the third most frequently detected hrHPV type in cervical cancers^10^. Furthermore, HPV35 is the most common hrHPV among women in rural Zimbabwe (19/648, 2.9%)^11^, and is the fifth most frequently identified hrHPV type in cervical cancers in Zimbabwe^12,13^. While specific genetic variants have been associated with carcinogenesis in other hrHPV types, HPV35 sequence data is relatively limited^1,2,14–16^. The present study describes 8 complete HPV35 whole genome sequences from self-collected cervical swab specimens obtained from women in rural Zimbabwe.

## Results

HPV35 whole genome sequences were obtained from previously collected cervicovaginal exfoliated cells from a study of hrHPV distribution in rural Zimbabwe^11^. HPV35 represented the largest proportion of HPV infections in that cohort; 2.9% of women (19/648) provided self-collected cervical swabs testing positive for HPV35. Of these 19 samples, mixed infections were excluded in order to evaluate for HPV35 variant associations with cytologic testing, and only available samples were extracted and sequenced (N = 9). Whole HPV35 genomes were successfully sequenced from 8 samples.

### Whole genome sequence analysis

**Figure 1:**
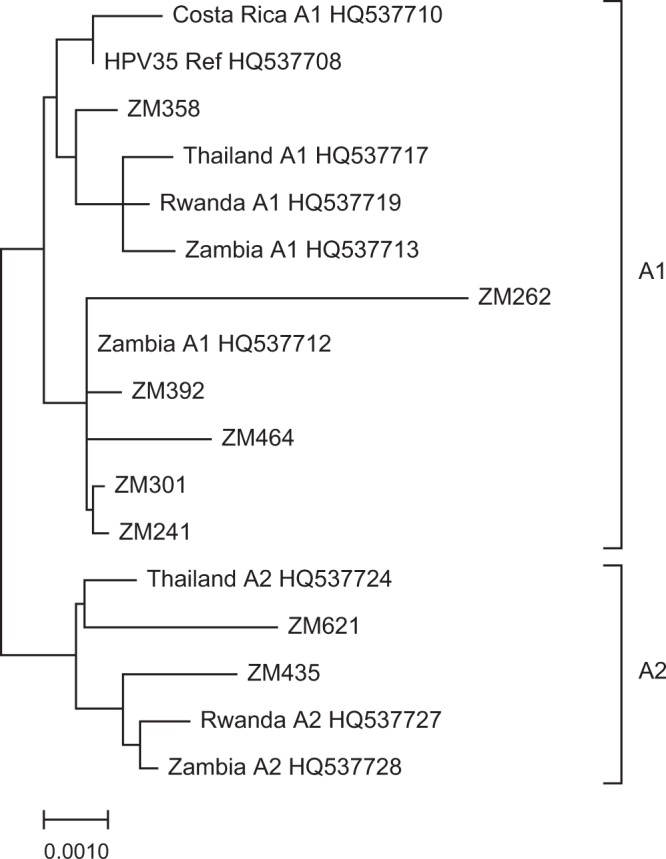
HPV35 whole genome phylogenetic tree. The phylogenic tree was generated using HPV35 whole genomes from Zimbabwe (ZM241, ZM262, ZM301, ZM358, ZM392, ZM435, ZM464, ZM621). The HPV35H reference sequence and previously reported HPV35 whole genome sequences were labelled with their country of origin and GenBank accession numbers.

Figure 1 shows the complete phylogenetic tree for the eight Zimbabwean samples when compared to the HPV35H reference, as well as representative A1 and A2 sublineage sequences from different geographic locations. Six samples are best classified as sublineage A1, while two are consistent with sublineage A2. The maximum number of nucleotide sequence variants compared to the prototype was 58 (58/7879; 0.7%), and the remainder of the sequences varied by 0.5% or less.

Of these successfully sequenced, HPV35–positive women, 3 (3/8, 37.5%) were HIV seropositive. There were no significant differences in HIV seropositivity or duration of antiretroviral therapy and HPV35 variants or carcinogenicity. Cytologic testing with liquid-based Pap smears was conducted concurrently with VIAC. Cytology results were obtained for 37.5% (3/8) of the HPV35 samples sequenced, including 1 negative for intraepithelial lesions or malignancy (NILM) and 2 high-grade intraepithelial lesions (HSIL; ZM435 and ZM621). VIAC was considered “precancer” for the two HSIL cases, as well as “precancer” for the sample considered negative by Pap cytology analysis. Both A2 samples were associated with HSIL.

### E6 gene

E6 gene variants are described in Table 1. The variants 127T > C, 136T > C, and 341T > C, have previously been identified^14,16^. A newly identified synonymous variant, 193G > A, was found in one sample tested, ZM392. The maximum number of nucleotide sequence variants compared to the E6 prototype was 3 (3/450, 0.7%).

**Table 1:** HPV35 E6 nucleotide variants.

Reference	127	136	193*	341
	T	T	G	T
ZM241	**C**	**C**	G	**C**
ZM262	**C**	**C**	G	**C**
ZM301	**C**	**C**	G	**C**
ZM358	T	T	G	T
ZM392	**C**	**C**	**A**	**C**
ZM435	**C**	**C**	G	**C**
ZM464	**C**	**C**	G	**C**
ZM621	**C**	**C**	G	**C**
Codon	78			
AA change	Trp78Arg	None	None	None

AA: Amino Acid; Trp: Tryptophan; Arg: Arginine. Based on reference sequence HPV35 HQ537708.1.

*Newly described variant.

### E7 gene

E7 gene variants are provided in Table 2. These variants were 675T > C and 682T > C. The maximum number of nucleotide sequence variants compared to the E7 prototype was 2 (2/300, 0.7%). Two samples, ZM435 and ZM621, had the same previously described synonymous variant (675T > C) associated with sublineage A2^2,14^. A newly described 682T > C variant was also found in sample ZM435, as well as ZM464. This non-synonymous variant results in a Gly41Cys amino acid change.

**Table 2:** HPV35 E7 nucleotide variants.

Reference	675	682
	T	G
ZM241	T	G
ZM262	T	G
ZM301	T	G
ZM358	T	G
ZM392	T	G
ZM435	**C**	**T**
ZM464	T	**T**
ZM621	**C**	G
Codon		41
AA change	None	Gly41Cys

AA: Amino Acid; Cys, Cysteine; Gly, Glycine.

Based on reference sequence HPV35 HQ537708.1.

### LCR polymorphism

LCR variants are shown in Table 3. These variants were 7156C > A, 7215T > G, 7261A > C, 7394A > C, 7535T > C, 7894C > A, and 7902G > A. A previously described 16-bp insertion was identified in 8 of 8 sequences^15,16^. The maximum number of nucleotide sequence variants compared to the prototype was 4 (4/770, 0.5%). Variants 7261A > C, 7394A > C, and 7535T > C have been previously described^14–16^ and occurred frequently in these sequences. Newly described variants include 7156C > A, 7215T > G, 7894C > A, and 7902G > A.

**Table 3:** HPV35 LCR nucleotide variants.

Reference	7156*	7215*	7261	7394	7535	7894*	7902*
	C	T	A	A	T	C	G
ZM241	C	T	**C**	**C**	**C**	C	G
ZM262	C	**G**	**C**	**C**	**C**	C	G
ZM301	C	T	**C**	**C**	**C**	C	G
ZM358	C	T	A	A	T	C	G
ZM392	C	T	**C**	**C**	**C**	C	G
ZM435	**A**	T	A	A	**C**	C	**A**
ZM464	C	T	**C**	**C**	**C**	C	G
ZM621	C	T	A	A	**C**	**A**	**A**

Based on reference sequence HPV35 HQ537708.1.

*Newly described variants.

## Discussion

This is one of the few studies examining genetic polymorphisms throughout the whole genome of HPV35, the most common hrHPV type identified in a community-based study conducted in rural Zimbabwe from 2016–2017^11^. Importantly, HPV35 is frequently identified in women with cervical dysplasia and cervical cancers in numerous studies in Southern sub-Saharan Africa^8–13,15–19^. Consistent with the literature, the data presented here demonstrates that HPV35 genetic variation is low relative to the variation observed in other hrHPV types^2,14–16,20–23^. In this study, the maximum nucleotide sequence variation compared to the prototype whole genome was 0.7%, and the remainder of the whole genome sequences varied by 0.5% or less. The limited variation observed in HPV35 may represent recent divergence from the most recent common ancestor of the HPV16, HPV31, and HPV35 clade^2^.

Consistent with the low level of genetic variation, HPV35 is divided into two sublineages, A1 and A2, rather than separate variant lineages, which are defined as requiring greater than 1.0% difference between complete genomes^2,24^. Other work suggests that HPV35 sequences may also fall into two evolutionary categories based on the presence or absence of the 16-bp insertion in the LCR^15,16^. This insertion is absent in the HPV35 reference sequence isolated in the United States but is present in all of the rural Zimbabwean whole genome sequences in this study.

While the association of the LCR insertion with the risk of viral persistence or dysplasia has not yet been investigated, several studies have evaluated the association of HPV35 sublineage with the risk of cervical intraepithelial neoplasia (CIN). For example, work from Costa Rica suggests that the A1 sublineage may be associated with higher risk of persistence and the development of CIN3+ than the A2 sublineage^25^. In contrast, a study from the United States identified no difference between HPV35 sublineages in the risk of CIN2/3^26^. Of the whole genomes sequenced herein, the two samples associated with HSIL by cytology were in the A2 sublineage. Future large scale studies including HPV whole genome sequencing will be required to determine whether the risk of dysplasia and cervical cancer are associated with HPV35 sublineage, and/or individual viral variants, including single nucleotide changes and insertion/deletions.

While HPV35 single nucleotide sequence variants have been previously reported, particularly in the E6, E7, and LCR genomic regions, the attribution of increased dysplasia or cervical cancer risk to particular variants has not been described^2,14–16^. In this study both previously described and newly described single nucleotide variants were identified. The newly described variants in these Zimbabwean viruses included E6 (193G > A), LCR (7156C > A, 7215T > G, 7894C > A, and 7902G > A), and a non-synonymous variant E7 (682T > C; Gly41Cys). Gagnon *et al*. suggest that HPV35 persistence may associated with the presence of non-synonymous changes in E7, though given the small number of sequences and participants there was insufficient power to investigate individual variants^14^. The study presented here was not designed to evaluate persistence, and future studies will be necessary to determine the role of this and other variants in persistent infection. Amino acid changes in HPV35 E7 could impact interactions with the retinoblastoma protein (Rb)^14^. Similarly, E6 amino acid changes may alter regulation of p53, whereas nucleotide changes in the LCR may change transcription factor binding. Once risk-associated individual variants are identified, functional experiments investigating their mechanism of action will be required.

## Conclusions

This study presents analysis of whole genome HPV35 sequences from self-collected cervical swab specimens obtained from women in rural Zimbabwe. There was an overall low level of nucleotide variation from the prototype HPV35 whole genome sequence. Newly described synonymous and non-synonymous single nucleotide variants were identified in E6 (193G > A), E7 (682T > C; Gly41Cys), and the LCR (7156C > A, 7215T > G, 7894C > A, and 7902G > A). Further studies are needed to establish whether these variants are associated with the development of cervical dysplasia and cervical cancers given the prevalence of HPV35 in Southern Africa and uncertain coverage by available HPV vaccines.

## Methods

### Sample collection and participant information

This study was nested under a previously reported cohort in rural Zimbabwe. Samples from the Chidamoyo study were selected if they tested positive for HPV35 by the Anyplex II HPV28 (Seegene Inc., Seoul, Korea) genotyping assay. Detailed study design and sample collection data were previously described.

HPV typing results and cytologic diagnoses of cervical lesion and characteristics of study subjects were obtained from the prior study. The protocol for the present study was approved by the institutional review boards at the University of Zimbabwe, Stanford University, Medical Research Council of Zimbabwe, and the Research Council of Zimbabwe.

### Sequencing methods

Viral nucleic acids were extracted from 200 µL of thin-prep samples using The EZ1 Virus Mini Kit v2.0 (Qiagen, Germantown, MD) and eluted in 60 µL. The whole region of HPV35 from each patient was amplified using primer pairs AF/AR and WF/DR (Table 1). Each reaction included LongAmp Hot Start Taq 2X Master Mix (New England Biolabs, Ipswich, MA), 400 nM of each primer, 3% DMSO and 5 µL nucleic acid. The reactions were run in a DNA Engine PTC-200 (Bio-Rad, Hercules, CA), with cycling conditions: 94 °C for 2 min; 40 cycles of 94 °C for 10 sec, 55 °C for 30 sec, and 65 °C for 5 min; final extension for 10 min at 65 °C. PCR products were sequenced by bi-directional dideoxynucleotide termination sequencing using AF, AR, WF, DR, EF, GF, KF, CR, MF, PF, and QR primers (Elim Biopharmaceuticals, Inc.; Hayward, CA). Primer sequences can be found in Table 4. The HPV35H (GenBank HQ537708) was used as the reference sequence. The individual sequences were aligned to the reference sequence using NCBI blast and the consensus sequences were generated from the assembly. The consensus sequences were manually curated by referring to the raw chromatograms where the base calls from two or more Sanger sequences showed conflict. Frame shifting insertion and deletions at nucleotide positions covered by a single sequence were discarded.

**Table 4:** Amplification and sequencing primers.

Name	Sequence (5′- to 3′)	Position^a^
AF	CCAGCTGGACAAGCAAAACC	685–704
AR	GAGACCCTGGAATAGGCGTG	4865–4846
WF	CCCACCTACAACAGGTTTTACA	4600_4621
DR	TTTGAAATGGCCTGCGTTGG	2913–2894
EF	TCTCAAGGACGTGGTGCAAA	2663–2689
GF	AGACGGGGACATGGTAGACA	6188–6207
KF	CTGCAGACTTAGATCAATTTCCGT	6961_6984
CR	TCCATTACATCCCGTCCCCT	918–899
MF	TATATGGAGCACCAAACACAGG	2249–2270
PF	AGGGCTTTAATTGCACACTTGGCTTTAC	7861–7705
QR	CTTTCGTTTTAGGACCTGTACAGC	1134–1111

^a^Based on HPV35 HQ537708.1.

### Construction of phylogenetic tree

The consensus sequences and a subset of HPV35 A1 and A2 sublineage sequences^2^ were subjected to multiple sequence alignment with default parameters using Clustal X (version 2.0)^27^. The phylogenetic tree was calculated from the multiple sequence alignment using the Neighbor-Joining algorithm in Clustal X, and the phylogenetic tree was then generated with Mega X^28^.

### Ethical approval and informed consent

Ethical approval was granted by Stanford University (#37975), University of Zimbabwe (JREC 221/16), the Medical Research Council of Zimbabwe (MRCZ/A/2128), and the Research Council of Zimbabwe (No. 02921). In addition, the Provincial and District Medical Officers were notified, as well as headmen and villages during community meetings, after sensitization via training of community health workers prior to data collection. Women were informed that their participation was voluntary, they could withdraw at any time, that we would offer to test for HIV but they could refuse this testing or refuse to be notified of their result, and that all information regarding their HIV and HPV status would be kept confidential. Most women wanted to know their HIV and HPV results. Eligible women were interviewed verbally by trained research data collectors on the research team using an electronic questionnaire to collect information on sociodemographic and reproductive information. Inclusion took place after individual informed consent signed electronically or with a thumbprint on a paper copy if illiterate, and an additional paper copy was given to the participation. Informed consent (signature or witnessed thumbprint) was obtained from all participants prior to enrolment. The methods were carried out in accordance with the relevant guidelines and regulations.

## Data Availability

The whole genome sequences described in this study are available in GenBank (Study Number, GenBank Accession Number): ZM241, MN829875; ZM301, MN829876; ZM464, MN829877; ZM392, MN829878; ZM358, MN829879; ZM435, MN829880; ZM621, MN829881; ZM262, MN829882.
